# MouseVUER: video based open-source system for laboratory mouse home-cage monitoring

**DOI:** 10.1038/s41598-024-52788-9

**Published:** 2024-02-01

**Authors:** Ghadi Salem, Niall Cope, Marcial Garmendia, Alex Pu, Abhishek Somenhalli, Jonathan Krynitsky, Noah Cubert, Thomas Jones, George Dold, Anthony Fletcher, Alexxai Kravitz, Thomas Pohida, John Dennis

**Affiliations:** 1 Instrumentation Development and Engineering Application Solutions, National Institute of Biomedical Imaging and Bioengineering, National Institutes of Health, Bethesda, MD USA; 2 Oak Ridge Institute for Science and Education (ORISE), US Department of Energy, Oak Ridge, TN USA; 3 Section On Instrumentation, National Institute of Mental Health, National Institutes of Health, Bethesda, MD USA; 4 Scientific Information Office, National Heart Lung and Blood Institute, National Institutes of Health, Bethesda, MD USA; 5 Dept of Psychiatry, Washington University in St Louis, St Louis, MO USA; 6 Division of Veterinary Services, Center for Biologics Evaluation and Research, US Food and Drug Administration, Silver Spring, MD USA

**Keywords:** Behavioural methods, Engineering

## Abstract

Video monitoring of mice in the home-cage reveals behavior profiles without the disruptions caused by specialized test setups and makes it possible to quantify changes in behavior patterns continually over long time frames. Several commercial home-cage monitoring systems are available with varying costs and capabilities; however there are currently no open-source systems for home-cage monitoring. We present an open-source system for top-down video monitoring of research mice in a slightly modified home-cage. The system is designed for integration with Allentown NexGen ventilated racks and allows unobstructed view of up to three mice, but can also be operated outside the rack. The system has an easy to duplicate and assemble home-cage design along with a video acquisition solution. The system utilizes a depth video camera, and we demonstrate the robustness of depth video for home-cage mice monitoring. For researchers without access to Allentown NexGen ventilated racks, we provide designs and assembly instructions for a standalone non-ventilated rack solution that holds three systems for more compact and efficient housing. We make all the design files, along with detailed assembly and installation instructions, available on the project webpage (https://github.com/NIH-CIT-OIR-SPIS/MouseVUER).

## Introduction

Video monitoring in mouse home-cages provides means of non-invasively capturing the animals’ natural behavior, including nocturnal behavior. Interest in home-cage video monitoring systems for mice has increased in recent years, and their utility is thoroughly documented in the literature. Several lab-specific systems have been presented in the literature^1^, however they are not suited for wide-scale adoption across different laboratories^1–6^. The scientific community has relied on a few commercially available systems for automated home-cage monitoring^1^. A recent entrant to the commercial home-cage monitoring systems market, one not included in Voikar and Gaburro’s work^1^, is Vium [Vium, San Mateo, CA]. Vium is set apart from all the other systems in that it offers a comprehensive ventilated rack and cage system specifically designed to facilitate video monitoring. All the commercial systems are based on conventional 2D video, and all have associated processing software with different capabilities. While all systems detect locomotive profiles, some also detect fine behaviors^1^. Despite their capabilities and accessibility, the high cost of commercial systems limits their widespread use in research. Additionally, proprietary algorithms can hinder reproducibility of results in other laboratories that are not in possession of the same commercial system. An open-source home-cage monitoring system along with open-source video analysis tools would facilitate the reproducibility of research results while eliminating the high cost of commercial systems. In the recent past, many open-source tools for automated locomotor and behavior analysis have been developed^2,6–8^. The field, however, still lacks an open-source scalable enclosure design with associated video acquisition methods.

High-capacity racks with individually ventilated mouse cages are the standard for most research animal care programs around the world, although large-scale automated monitoring has not yet been adopted despite the potential high impact and utility. One reason for this is that video monitoring is greatly hindered by rack and cage obstacles. The source of the challenge is that the ventilated racks were mainly manufactured prior to the ubiquity of video acquisition and analysis technology, and hence no consideration was given to incorporation of video monitoring. Due to the design variety in commercial racks and cages, a system made for integration with a legacy rack will work solely with the rack for which it was designed. Two systems were designed to integrate video monitoring into legacy ventilated racks. One is Actual Analytics^9^, a commercial product specific to Tecniplast racks [Tecniplast Inc., Buguggiate, Italy]. The other is SCORHE^4^ designed for Thoren racks [Thoren Inc., Hazleton, Pennsylvania]. SCORHE is not commercial, however it has an intricate design that is difficult to replicate. Furthermore, the system utilizes fisheye lenses that compromise processing accuracy. Vium is the first company to have a forward-thinking approach to large-scale video monitoring in ventilated racks. The company designed the rack and cages around the video monitoring equipment.

In order to address the need for an open-source enclosure design with integration into a ventilated rack [Allentown Inc, Allentown, NJ], we developed the Mouse Video Utility for Experiments Recording (MouseVUER, pronounced ‘Mouse Viewer’). The hardware for this scalable system is easy to fabricate and assemble. Duplicating the system would require access to prototyping tools such as 3D printers and laser cutters. MouseVUER utilizes an IntelSense D435 depth camera [Intel Inc., Santa Clara, California] with options for streaming with RGB, depth, and near-infrared video simultaneously. The system also comprises video acquisition tools for streaming and storing video. The system can be used in Allentown NexGen ventilated rack [Allentown Inc, Allentown, NJ]. However, our open-source solution also includes a custom 3-unit rack design. The custom rack offers space efficient housing for three systems along with cable management.

## Materials and methods

### Animal activities

Mice were housed together in individually-ventilated cages (Allentown NexGen500) with one to five mice per cage. All mice were maintained on a regular diurnal lighting cycle (12:12 light:dark) with room temperature maintained at 72 F ± 1° and humidity 30–70%. Environmental enrichment includes nesting material (Bed-r Nest, The Andersons Inc), plastic tunnel (Datesand Limited), and cardboard tunnel (Pakolatus, LLC). Mice were regularly provided edible enrichment as forage (Fruit and Veggie Medley, Irradiated, Bio-serve). Handling was by both tunnel transfer and tail restraint. Mice were housed under specific pathogen-free conditions in the White Oak Animal Program vivarium of The Food and Drug Administration accredited by AAALAC International. The entire study was consistent with the ARRIVE Guidelines, and approved by FDA’s White Oak Animal Program IACUC. All the research and activities were carried out in accordance with the guidelines and regulations.

Genetically-engineered mice of multiple background strains, ages, and coat colors and both genders were used for optimizing and testing at these stages of monitoring for hardware optimization. For monitoring sessions in the MouseVUER system, individual mice were removed from social groups for overnight recording and returned to familiar social groups following data collection. Strain names included Balb/c, C57Bl/6 B6-IFN-beta KO, and B9-FPR-1,2 F2.

### Mechanical design

All MouseVUER component CAD models were designed in SolidWorks [Dassault Systèmes SolidWorks Corporation, Waltham, MA]. Acrylic panels were cut from ¼” thick clear bulk material [P/N: Cast Acrylic Sheet – ¼” × 24″ × 24″ Clear, Elkridge, MD] using a CO_2_ laser cutter [PLS6.150D, Universal Laser Systems]. Parts were 3D printed from micro carbon fiber filled nylon filament [Onyx, Markforged] using a FDM printer [X7, Markforged]. Stainless Steel pieces were cut from bulk material using a waterjet cutter [OMAX 2626, Omax Corporation, Kent, Washington]. All the design files, along with detailed fabrication and assembly instructions, are available on the project page (https://github.com/NIH-CIT-OIR-SPIS/MouseVUER).

## Results

### Design and construction of the apparatus

#### Modified NexGen lid

**Figure 1:**
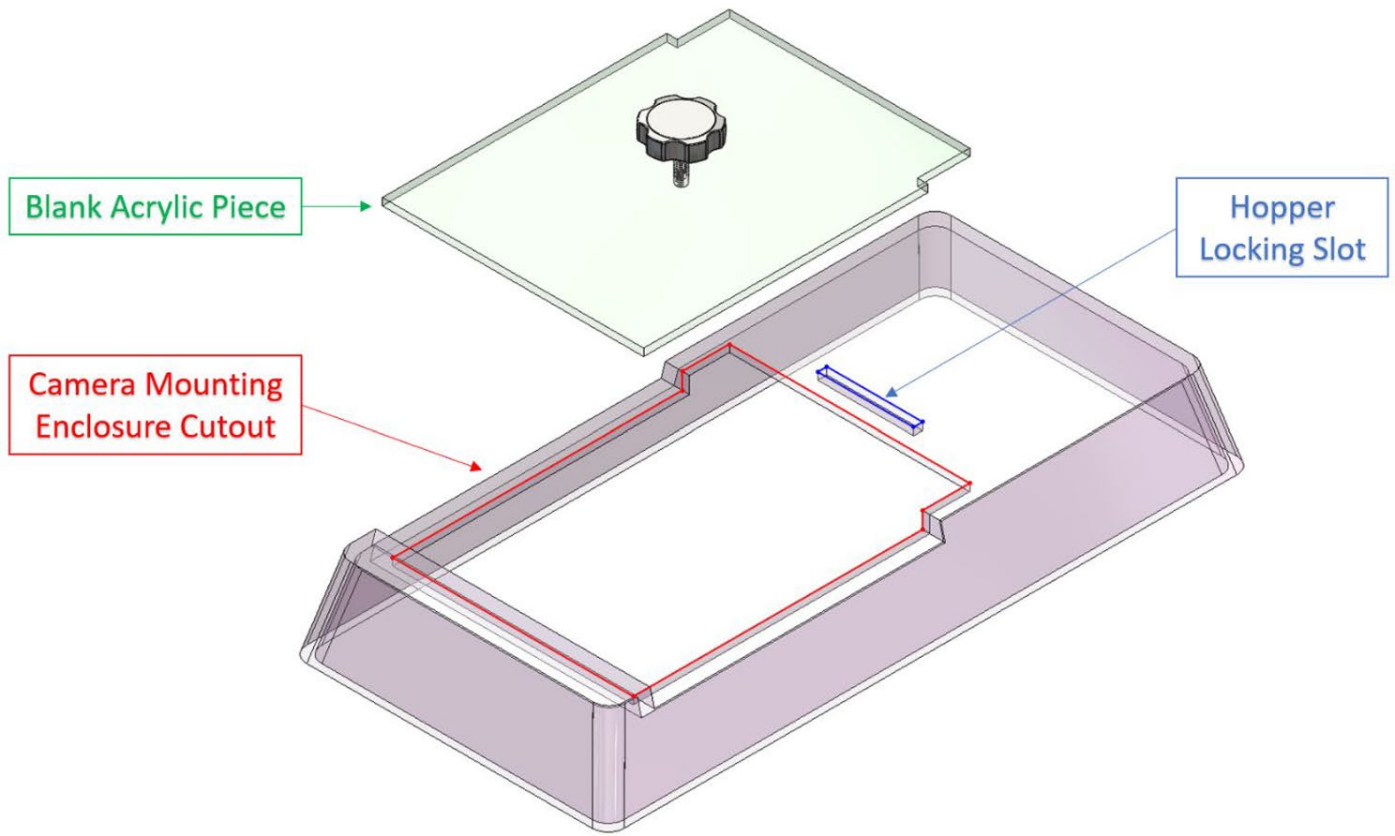
Modified Allentown Lid– Cutouts were made on commercially available Allentown lids to lock the hopper in place and to provide an unobstructed camera view. A cut-to-shape acrylic piece is placed on the lid when the camera mounting enclosure is not used. The image was generated with SolidWorks Visualize 2022 and annotated with Microsoft PowerPoint.

**Figure 2:**
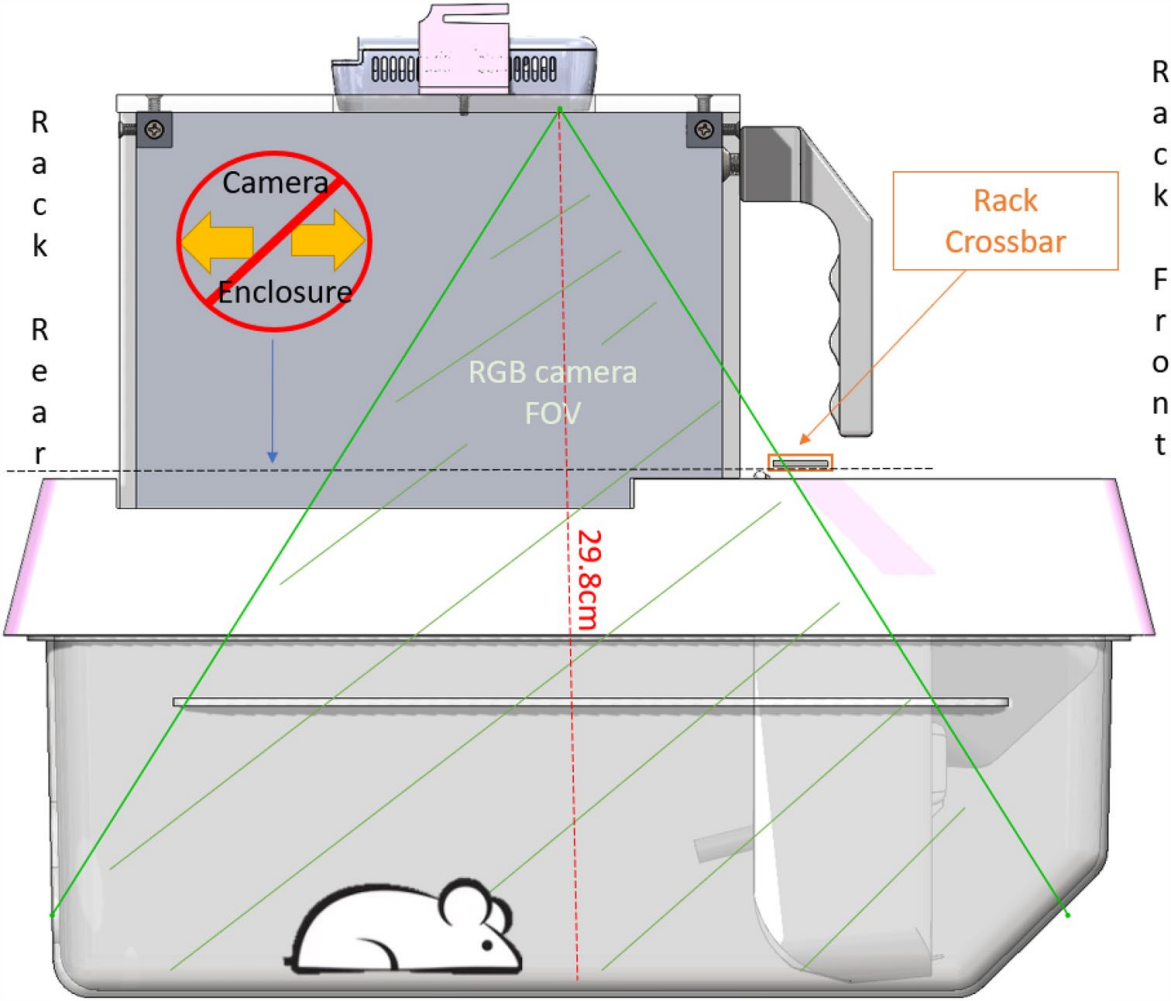
MouseVUER system – Camera mounting enclosure over modified Allentown lid and Cage. The camera is positioned to capture an unobstructed view of the mouse. Due to a crossbar at the front of the rack, the camera mounting enclosure must be placed on top of the cage AFTER it has been inserted/slid into the rack. The image was generated with SolidWorks Visualize 2022 and annotated with Microsoft PowerPoint.

To reduce design and manufacturing costs, an existing Allentown NexGen lid was modified to mount the camera system. Allentown offers multiple lid options for use with the NexGen cages. The solid clear lids [P/N: 228,790–1, Allentown Inc, Allentown, NJ] were selected for use. The lid has a clear solid top, as opposed to the other options which have filter paper on the top that blocks the cage from the camera view. Even though the lid is made of clear acrylic, the acrylic compromised the depth image quality. The solution we found to preserve image quality was to have no barrier at all between the camera and the cage arena. Hence, a cutout was made on the lid using a waterjet cutter to provide an unobstructed line of sight for the camera as illustrated in Fig. 1. The cutout was a patterned cut that mate with the camera mounting enclosure. In choosing the dimensions of the cutout and its position in the lid, we considered the presence of a cross bar in the Allentown rack that would obstruct a portion of the cage from the camera view as seen in Fig. 2. Hence, we chose to restrict the usable cage floor space and therefore the lid cutout to approximately the back two thirds of the cage. In the front third of the cage, we placed the food hopper and water bottle basket. Except for the cage-facing surface, the food hopper is not visible through the camera. Only the area of the hopper accessible to the mouse appears in the image. When the camera mounting enclosure is placed on the lid, the cage becomes closed off such that the mice are not able to escape. When operating in an Allentown rack, however, the cage cannot be inserted into the rack with the camera mounting enclosure secured on the lid due to the cross bar at the front of the rack. Therefore, when inserting the cage into the rack or removing it from the rack, the camera mounting enclosure must be removed as presented in Fig. 2. When the camera mounting enclosure is removed, however, the cage top is mostly open. Therefore, to prevent mice from escaping, we also designed an acrylic piece to block the cutout in the lid while the cage is being moved as illustrated in Fig. 1.

#### Custom food and water hopper

**Figure 3:**
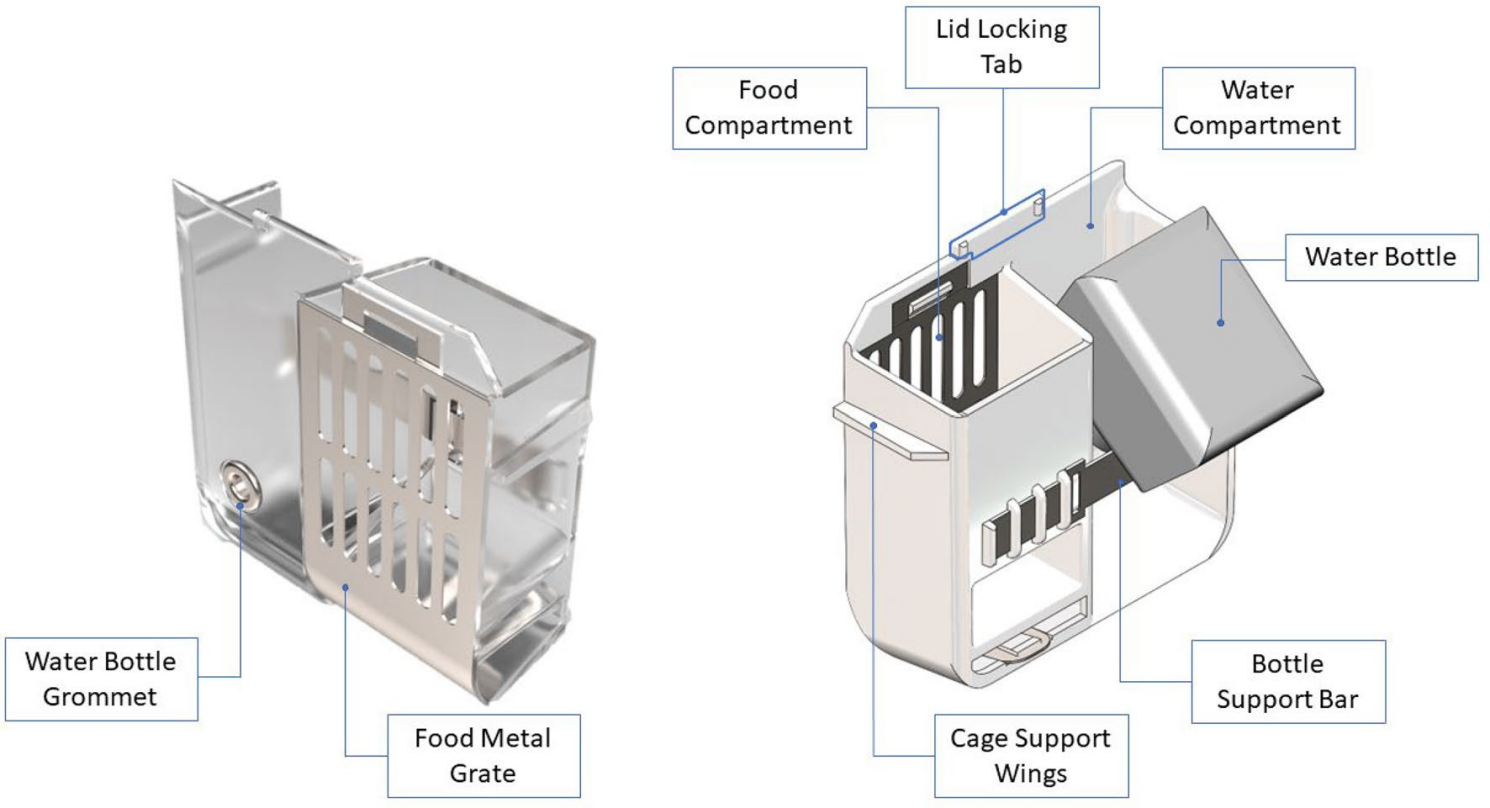
Food and Water Hopper – Custom 2-compartment hopper for food and water. The compartments are protected with metal grates at the front to prevent mice from chewing any edges. The cage support wings rest on the inner edge of the cage while the locking tab fits into a slot made in the modified lid. A commercially available water bottle is supported at an angle by the bottle support bar. The images were generated with SolidWorks Visualize 2022 and annotated with Microsoft PowerPoint.

A custom, two-compartment hopper was designed to house food pellets and a water bottle. The hopper was injection molded [ICOMold Inc., Hartland, WI] from a food safe, autoclavable clear material [PSU Ultrason S3010, BASF, Ludwigshafen, Germany] and was designed to fit inside a NexGen standard cage [P/N: 223,581–4, Allentown Inc, Allentown, NJ]. The hopper’s support edges rest on the cage’s top inner edge, and locks to the front of the cage via a tab-slot feature on the lid as illustrated in Fig. 3.

The food compartment has an internal volume of 378 cm^3^. A custom-made metal grate [304SS, 26-gauge, Yarde Metal, White Marsh, MD] is used to contain the food in the hopper while allowing the mice to eat through slotted holes. The sharp edges of the grate are deburred by placing the grate pieces in a tumbler. The grate is formed around an opening in the injection molded hopper and locks into the hopper using tabs. The grate is mated to the hopper exterior by a carefully designed lip, so it prevents the mice from chewing on the edges of the injection molded chamber.

The water compartment holds a 9 oz. water bottle [BW09UIRNO, Thoren Caging Inc., Hazleton, PA] with a sipper tube [BWCS250HSB, Thoren Caging Inc, Hazelton, PA]. The water bottle is supported at the rear by a custom-made support bar [304SS, 24-gauge, Yarde Metal, White Marsh, MD] that slides into the hopper. A grommet [304SS, trade size 2, Yarde Metal, White Marsh, MD], installed with a hammer-driven punch [9612K28, McMaster-Carr, Elmhurst, IL], protects the edges of the hopper cavity around the sipper tube.

We chose to place the hopper at the front of the cage. The reason for the choice of placement, as explained in the Modified NexGen Lid section, is to occupy the part of the cage partially obstructed from the view of the camera by the horizontal cross bar in the rack. While the content of the food basket is not visible through the camera, the metal grate is in full view. Hence, the mouse interaction with the food is in the field of view of the camera. Similarly, the water bottle itself is not visible through the camera, however the sipping tube is in the field of view. Hence, any interaction of the mouse with the water will also be captured by the camera. The tab on the hopper along with the mating slot on the lid offer a locking mechanism to fix the position of the hopper relative to the camera. Fixing the position of the hopper relative to the cage ensures a consistent environment for video acquisition because the placement is repeatable and ensures food and water consumption are always in clear view of the depth camera.

#### Camera mounting enclosure

**Figure 4:**
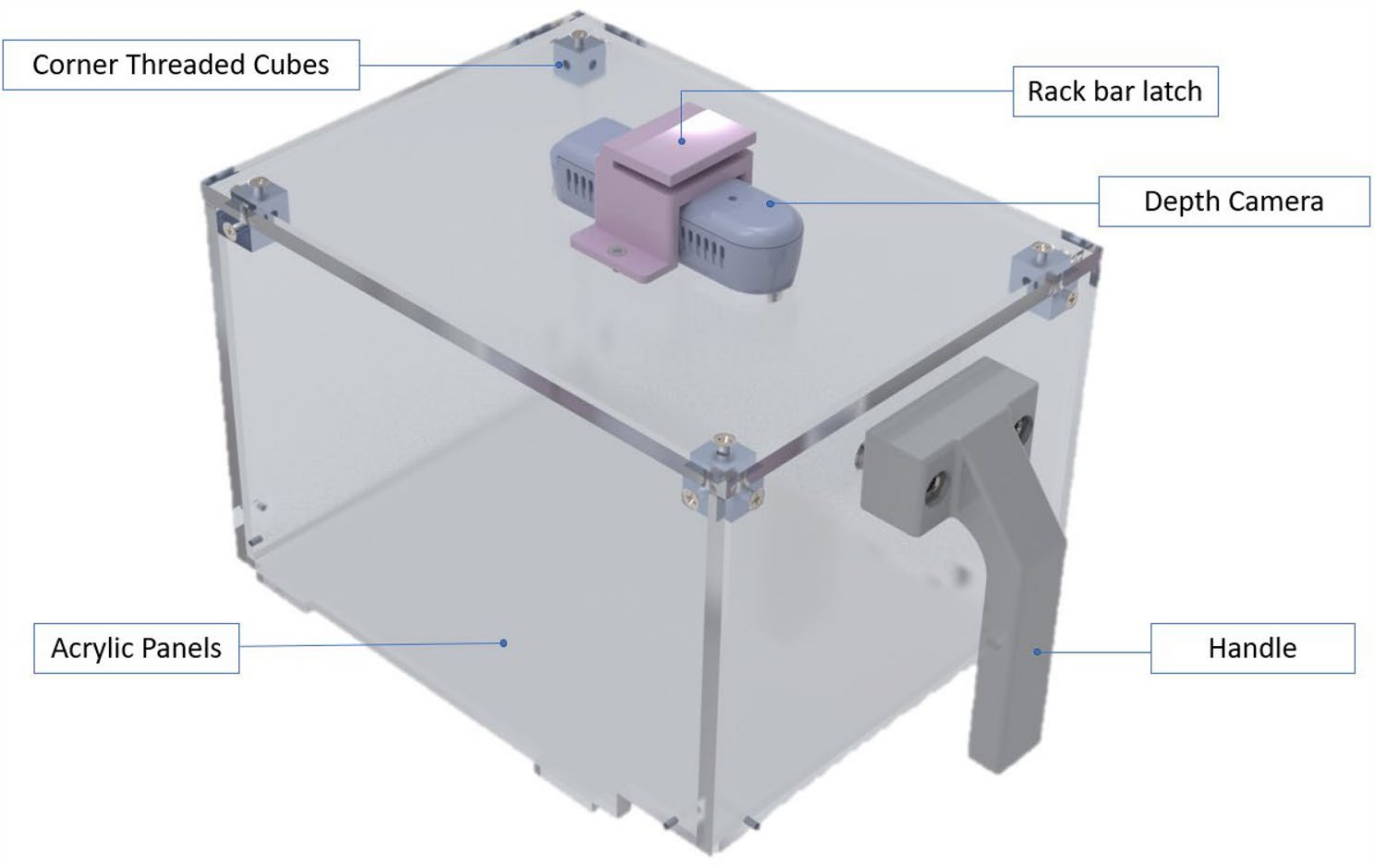
Camera Mounting Enclosure—Positions depth camera above cage environment for unobstructed video recording. The camera attachment (rack bar latch) latches to a crossbar above the cage during cage changes. A front handle allows for easy handling of the enclosure. The panels are made from clear acrylic to allow view of the mice to observers. The image was generated with SolidWorks Visualize 2022 and annotated with Microsoft PowerPoint.

The camera mounting enclosure houses the overhead video recording depth camera as illustrated in Fig. 4. Custom-cut acrylic panels, Fig. 1, joined at the top corners with threaded cubes [P/N: Aluminum M3 Cubes, Farmers Branch, TX], form a five-sided enclosure box with smooth internal walls to prevent mouse climbing [Clear Cast Acrylic-1/4″, Piedmont Plastics, Elkridge, Maryland]. A depth camera [Intel RealSense D435, Intel Inc., Santa Clara, California] is attached to the top panel via a custom 3D printed camera mount such that it sits 29.8 cm above the cage floor and captures an unobstructed view of all regions of interest. 29.8 cm was chosen to meet the minimum working distance required by the camera. Additionally, the camera mount includes a slot to hang the enclosure on a rack crossbar above the cage. Suspending the enclosure above the cage allows easy access to the cage and frees up the user’s hands to handle the cage (e.g., during cage changes). The enclosure has a custom 3D printed handle that is attached to the front panels via screws. When in use, the enclosure securely sits on a modified NexGen lid [P/N: 228,655–5, Allentown Inc, Allentown, NJ]. The enclosure consists of: laser cut acrylic panels, a 3D printed camera mount, a 3D printed handle, and aluminum threaded cubes.

#### Mezzanine

**Figure 5:**
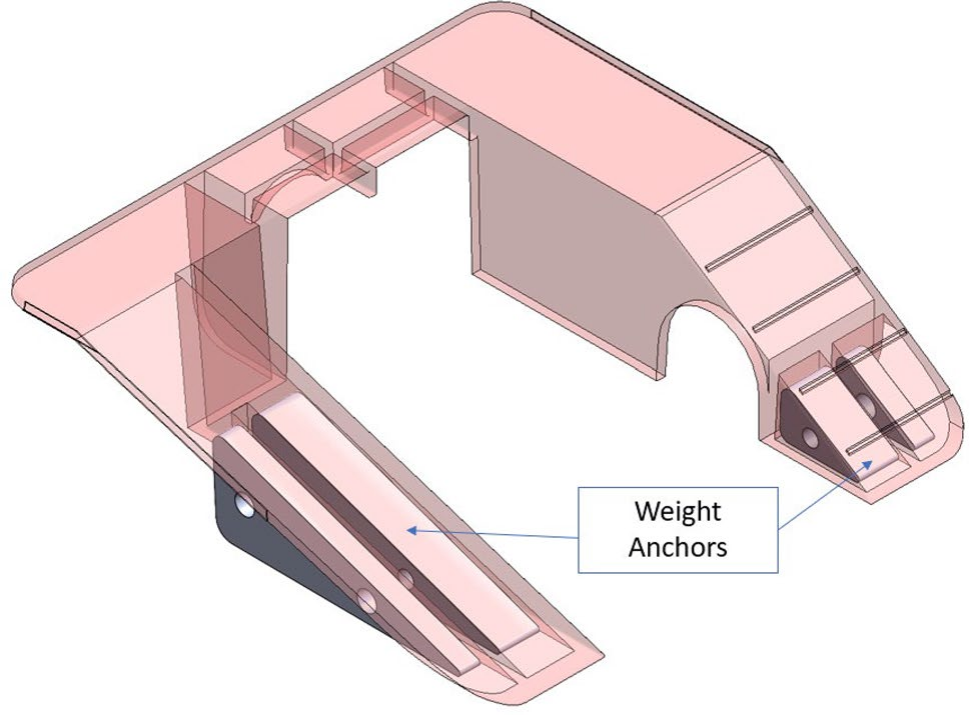
Mouse Mezzanine—Provides enrichment to the mice (ramps, tunnels, etc.). On the sides, weight anchors have been added to prevent potential dragging or flipping. The image was generated with SolidWorks Visualize 2022 and annotated with Microsoft PowerPoint.

The custom-designed mezzanine is a means of cage enrichment. The mezzanine offers an incline up to a flat surface for mice to walk on and a tunnel as seen in Fig. 5. The mezzanine is injection molded from a corrosion-resistant stainless-steel sheet [P/N: 9195K41, McMaster-Carr Inc., Elmhurst, IL] and fits inside a NexGen standard cage alongside the custom food and water hopper. The mezzanine was designed to have weight anchors to prevent mice from shifting or flipping it. Custom waterjet-cut metal triangular blocks [316 SS, ½” and ¼” thick] mount to the sides of the mezzanine via screws to weigh it down.

#### Non-ventilated rack

**Figure 6:**
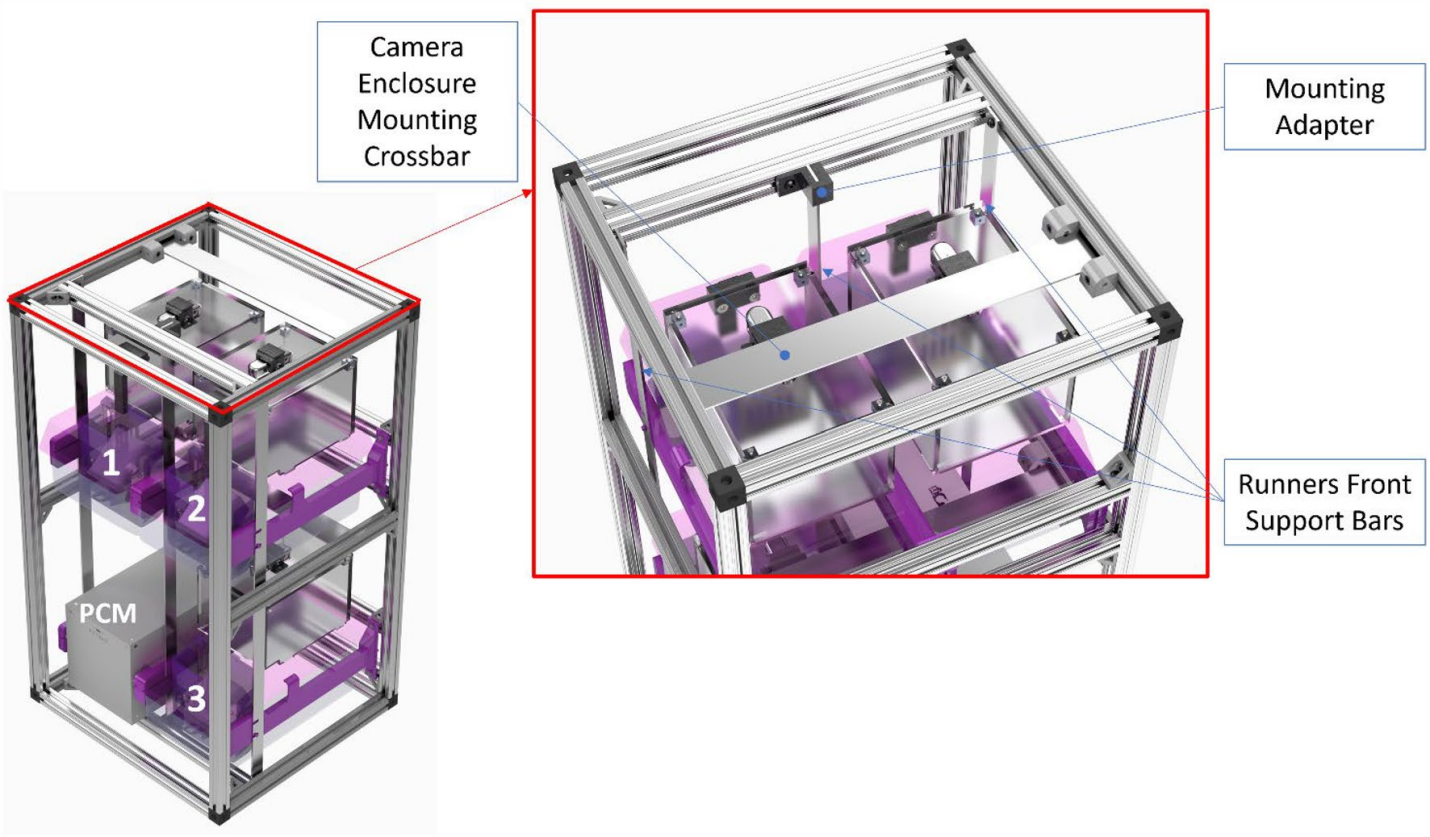
Custom Cage Racks—Modular Rack which houses three MouseVUER systems and associated electronics. The rack includes horizontal bars to hang the camera mounting enclosures during cage changes. Due to its modular properties, the rack can be expanded to fit any number of MouseVUER systems. The image was generated with SolidWorks Visualize 2023 and annotated with Microsoft PowerPoint.

The MouseVUER units can be operated on a benchtop. For more compact housing, we have designed a non-ventilated rack (Fig. 6) to hold three MouseVUER units, house associated electronics, and provide cable management. The modular frame design enables the MouseVUER to be used in a higher density setup for users who do not own OEM Allentown racks. The design allows for future expansion and stacking of units at a low cost. The rack frame is built using 80/20 T-slot aluminum bars (1″ × 1″ × 1″) and fasteners. Allentown OEM runners [P/N: K10000, Allentown Inc, Allentown, NJ] are used to support NexGen cages. The runner’s mount to the rear 80/20 bars using adjustable screws and use waterjet cut custom metal support bars (aluminum, $${\raise0.7ex\hbox{$1$} \!\mathord{\left/ {\vphantom {1 8}}\right.\kern-0pt} \!\lower0.7ex\hbox{$8$}}$$” thick) in the front. A custom metal crossbar [Aluminum, $${\raise0.7ex\hbox{$1$} \!\mathord{\left/ {\vphantom {1 8}}\right.\kern-0pt} \!\lower0.7ex\hbox{$8$}}$$” thick bulk material, P/N: 8975K582, McMaster Inc, Elmhurst, IL] mounts horizontally to hang the camera mounting enclosure. The nonventilated racks have 4 slots for cage placement.

**Figure 7:**
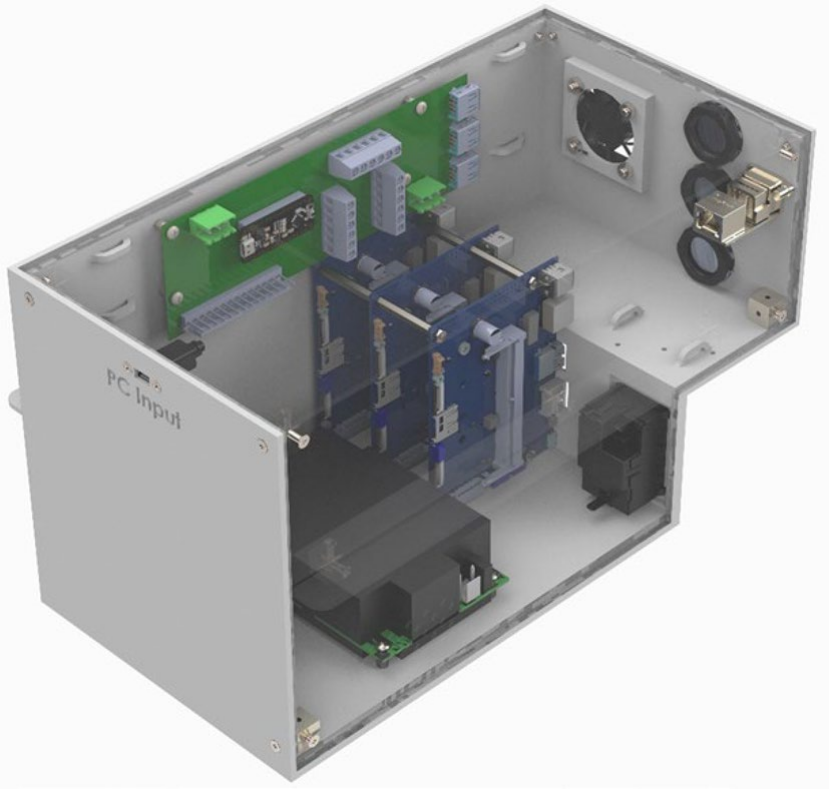
Power and control module which provides electronics for powering and establishing communication with three monitoring units. The power and control unit also has 3 auxiliary lines to power external illumination to cages if needed. The image was generated with SolidWorks Visualize 2023.

We designed an electronics enclosure, hereinafter referred to as the Power and Control Module (PCM), responsible for supplying powering to and data communication with three MouseVUER systems to successfully monitor the rodents (Fig. 7). The PCM is designed to slide into one of the open slots on the custom racks using 3D printed rails. Hence when using the PCM, the rack capacity is reduced to three cages. The PCM has electronic connections in the rear to the three MouseVUER systems. Each PCM has three single board computers (SBC) [70878-AI-TX2, Auvidea GmbH, Denklingen, Germany] utilized as compression units compressing video from one of the MouseVUER units and sending the compressed video via ethernet connection to a network switch [EH2306, ATOP Technologies, Zhubei City, Taiwan] inside the PCM. The switch relays the data to an ethernet panel mount [MRJ-5780–01, Amphenol ICC, Wallingford, CT] on the PCM such that the data can be streamed over a local network to a connected PC for storage. The PCM has a power supply [MPB125-4350G, Bel Power Solutions, New Jersey City, NJ] that connects to a power entry module [KM01.1105.11, Schurter Inc., Santa Rosa, CA] which can be powered by a standard AC 110 V wall outlet. The power supply is connected to a custom power distribution board responsible for powering the internal components of the PCM. The internal components powered by the power distribution board include the three SBC compression units, the ethernet switch, a fan [MF40100V1-1000U-A99, Sunon Fans, Brea, CA], and a microcontroller [2590, Adafruit Industries LLC, New York, NY]. The fan helps with thermal management by providing airflow through the PCM to prevent overheating of the internal components. The microcontroller allows for controlling auxiliary power options. The PCM has multiple connections in the rear. Two connections are for the power module and ethernet port. Three connections are for individual data communication between the PCM and each MouseVUER system. Three 6 pin connectors are available for auxiliary power for future capabilities such as controlling external LED lighting on each of the MouseVUER systems.

### Video acquisition solutions

As stated earlier, MouseVUER utilizes the Intel RealSense D435 Depth Camera for video recording. Intel offers a GUI application, called RealSense Viewer, for acquisition. The compression rates are not efficient, yielding approximately 1GB of data for each minute of recording from all 3 streams (i.e., depth, near-infrared, and RGB). Intel offers a software development kit (SDK) for the RealSense camera that enables development of software that interfaces with the camera. We developed a custom software specifically for MouseVUER based on the Intel SDK. Recordings can be scheduled by entering start/end times and the user’s presence is not required to manually initiate and halt recordings. The user can enter experiment metadata into the GUI. The GUI is derived from that described by Salem and colleagues^10^.

**Figure 8:**
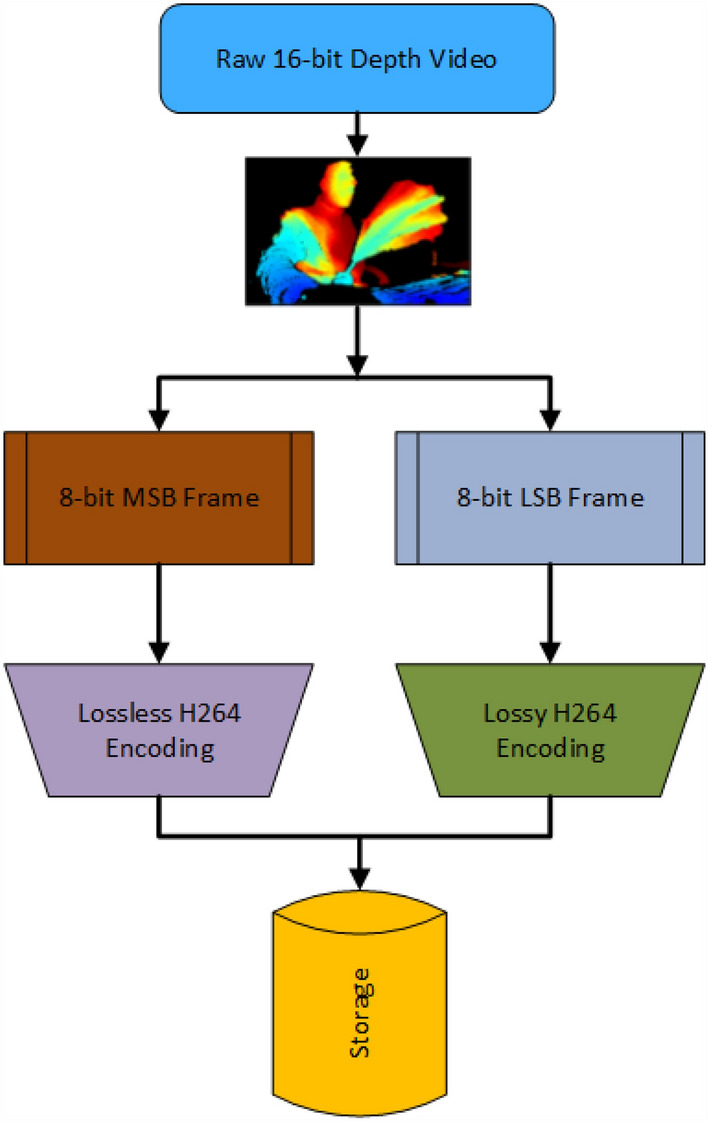
Compression Pipeline. A sequence of depth frames (colorized for visualization purposes) is split into 8-bit video frames. Each video frame is then compressed and sent to a central storage unit.

The most impactful feature of the custom acquisition software is the compression rate. Our compression method employs the open-source FFmpeg library for encoding 16-bit depth frames into video. The compression is done by splitting each 16-bit frame into two 8-bit frames, the most significant bit (MSB) frame and the least significant bit (LSB) frame (Fig. 8). The MSB frame is then compressed utilizing × 264 encoding. The LSB frame is encoded in a lossy manner by the × 264 encoder. The LSB frame stores the commonly changing data in the video. The MSB frame, however, changes infrequently, thus can be stored losslessly. We end up with two compressed video files that can be recombined to obtain the original depth data. The user can play back the recording to verify the data integrity. Our software also allows the simultaneous recording and streaming of RGB data, aligned depth video and infrared data from multiple computers to a central server. Aligned depth video is video where the depth camera is aligned with the coordinates of the RGB camera. This alignment process requires extra processing and can degrade performance if the range of depth values is too large. For mice recordings depth values between 0 and 4096 are sufficient. To use the full range (i.e., 0– 65,535), the alignment feature should be turned off. All options can be defined in the Intel RealSense software JSON file. We also provide a decompression tool so the user can recombine and obtain back original data.

**Figure 9:**
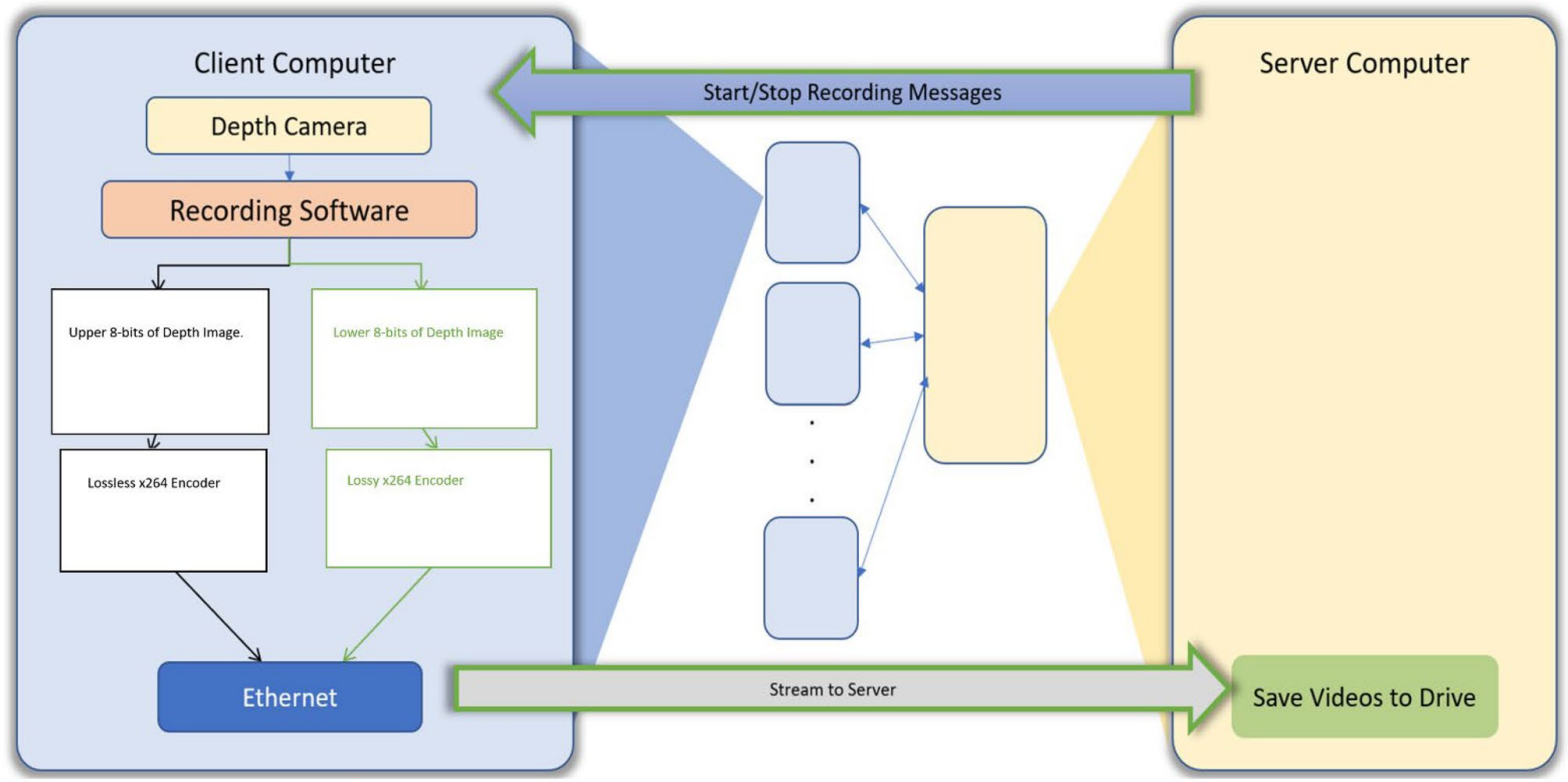
A visual diagram showing the general function of the server computer and client computer as well as the relation between the two. Multiple client computers (indicated by blue boxes) connect to a single server computer (indicated by yellow box).

Our software allows for simultaneous streaming to a central server from multiple computers (i.e., with a camera connected to each computer), with a GUI for starting and stopping recordings. For our client computers that house our depth cameras we chose to utilize HP EliteDesk 800 G2 Mini Business Desktop PC Intel Quad-Core i5-6500T equipped with 8GB of DDR4 RAM. The mini-desktops are cost and power efficient. The server computer has fewer computational restrictions and mainly serves to store video (Fig. 9).

Our software also allows for high compression and quality performance. To measure the performance of our compression we use Peak-Signal-to-Noise Ratio (PSNR) for video quality assessment and compression ratio for saved storage assessment. PSNR is computed using the equations:

$$ MSE = \frac{1}{m*n}\sum\nolimits_{x = 0}^{m - 1} {\sum\nolimits_{y = 0}^{n - 1} {\left( {I\left( {x,\;y} \right)_{orig} - I\left( {x,\;y} \right)_{comp} } \right)^{2} } } ;\;PSNR = 10\log_{10} \left( {\frac{65535}{{MSE}}^{2} } \right) $$

The method we designed was tested with different settings on a 5-min uncompressed depth only recording of mice. A 9 × compression ratio was achieved with 82 dB PSNR, and a 21 × compression ration was achieved with 65dB PSNR. The project Github page (https://github.com/NIH-CIT-OIR-SPIS/MouseVUER) has detailed instructions on setup and testing of our solution.

We note that users of the MouseVUER system can elect to use commercial options for video recording. Namely, we’ve utilized two software systems aside from our in-house developed software. The first is Intel’s RealSense Viewer, a GUI application based on the SDK that allows the user to connect multiple cameras simultaneously and preview a single camera stream. This software provides easy access to set and modify acquisition parameters. The chosen acquisition parameters can be stored in a configuration file which can then be loaded whenever the software is launched again. GUI controls allow the user to toggle between cameras and start/stop recording for each camera individually. Unfortunately, the Intel RealSense Viewer has limitations that especially impact its viability for home-cage monitoring studies. First, the compression rates are not efficient, yielding approximately 1GB of data for each minute of recording from all 3 streams (i.e., depth, near-infrared, and RGB). Considering that home-cage monitoring studies could span multiple circadian cycles, the volume of generated video data very quickly becomes impractical to store and transport. Second, the RealSense software lacks the ability to set an automated start and stop time for recording. Instead, the user’s presence is required to manually start and stop recordings. Third, the software offers no means of specifying experimental metadata. Lastly, the recordings are stored in a single file. File operations on a single large file might not be efficient, and it might be desirable to have the option to store recordings in clips of prespecified duration.

The second commercial video acquisition solution is a feature-rich, web-based streaming software package by Aivero [Aivero, Stavanger, Norway]. Aivero software allows for scheduling of recordings with multiple RealSense cameras used in tandem. Users can fully modify each recording session with the desired streams (depth, RGB, and near-infrared), schedule start times, set recording durations, select and preview the cameras from which to record, and split a single experiment into multiple smaller video clips of user selected maximum file size. The software also includes playback functionality for recorded videos. The Aivero platform relies on external hardware acceleration device for compression. Having the video compressed by a hardware acceleration device running the Aivero codec software allows for streaming at a much lower bandwidth either to a remote processing server or to a local host computer running the Aivero host software. The compression software was first implemented on JN30D-TX2 [Auvidea GmbH, Denklingen, Germany]. The acceleration device takes a USB 3.0 connection as input (i.e. from the camera) and outputs the compressed stream over ethernet. The compression is lossless for the depth data and lossy for the color and infrared data. A 7:1 compression ratio was achieved without visually affecting the quality of the image. This ratio varies depending on the complexity of the image. Aivero compression software can also run on other hardware acceleration platforms such as Nvidia Jetson Nano.

### Characterization of final system

Using the MouseVUER system, we acquired video from three cages, each holding a solitary housed mouse having a different coat color: black, white, and tan. The experiment was carried out with both depth and RGB streaming enabled, using the Aivero software. The goal of the experiment was to illustrate the robustness of the keypoints detection task on depth images.

**Figure 10:**
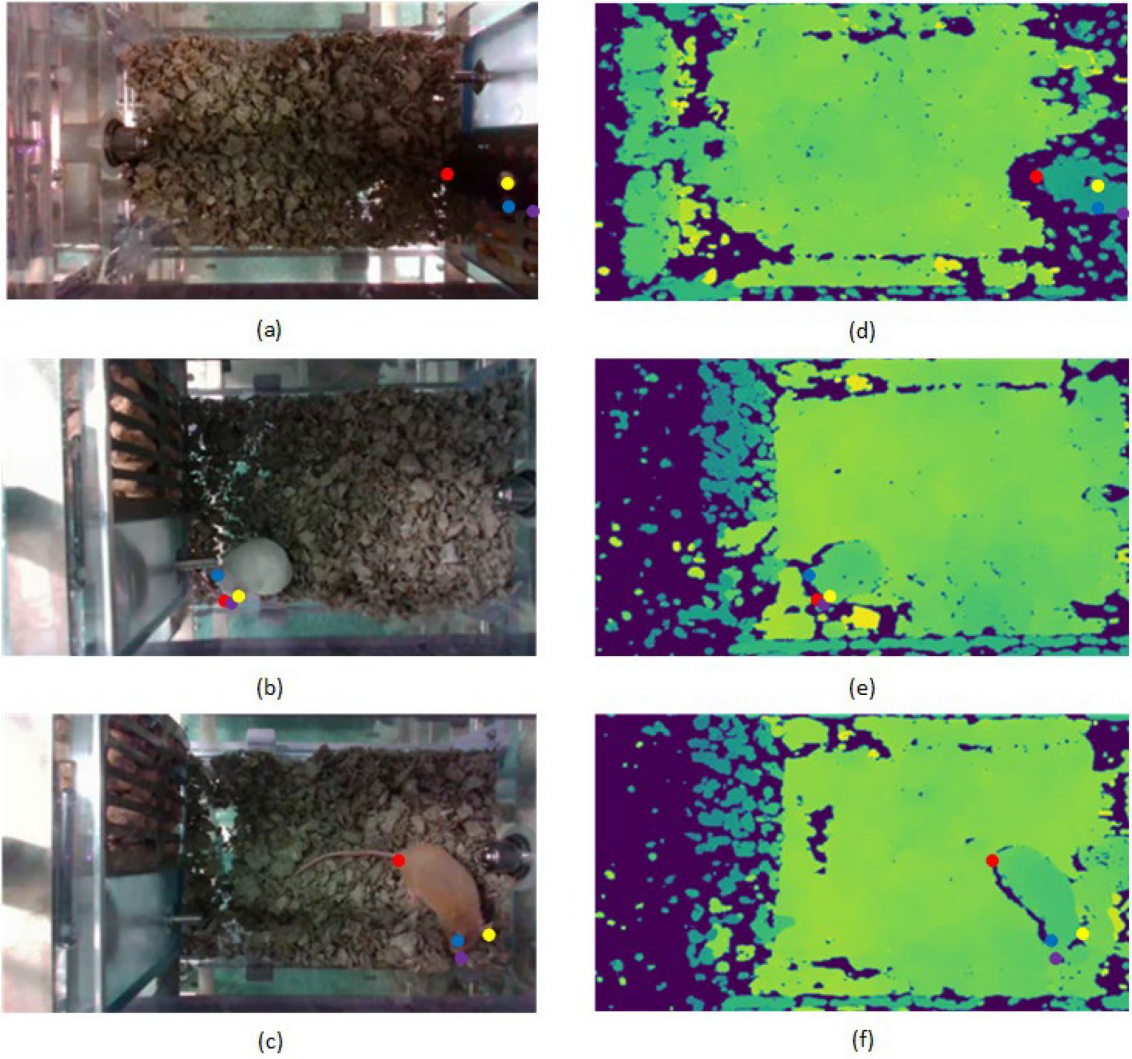
Example frames of an annotated mouse subject from (**a**–**c**) the color stream and (**d**–**f**) the depth stream. Body parts include nose, left ear, right ear, and tail base. Compared to the RGB stream, the depth stream provides a significant visual challenge for the annotation of the mouse’s key points for annotation tasks.

For keypoint detection, we made use of the popular open-source deep learning tool DeepLabCut (DLC)^2^, which provides systems for annotating data, training models, and finetuning results to reach acceptable levels of keypoint inference accuracy. For our purposes, four key points on a solitary housed mouse were annotated: nose, left ear, right ear, and tail base. Annotating directly on colorized depth images is not necessarily straight forward (Fig. 10). To facilitate the annotation task, we chose to record both RGB and depth videos simultaneously. We restricted annotations to RGB images from daylight recording since the room light was sufficient for the D435 to stream clear RGB video simultaneous with the depth stream. The RGB and depth frames, both acquired from the RealSense D435, were temporally aligned, as the streams were recorded simultaneously. Given the difference in frame size between color (1920 × 1080 pixels) and infrared/depth (1280 × 720 pixels), spatial alignment was conducted to fit the RGB frame onto the depth frame. The temporal alignment was done via Aivero software tools. In particular, the edge devices running the Aivero client software synchronize the clock of the recording pipeline with a Network Time Protocol (NTP) clock server running on the server computer. The NTP server configures the Real-Time Streaming Protocol (RTSP) connection to reconstruct meaningful timestamps on the server side, ensuring the data from multiple cameras and camera streams are synchronized. Furthermore, they store each camera's real-world start time in the recorded video files using the `Encoded date` file tag. The temporal and spatial alignment of RGB and depth images makes the annotations done on RGB images applicable to the corresponding depth images. Therefore, a depth image prediction model can be trained on the depth images with the same RGB annotations for the time matched RGB images. A set of 100 RGB frames along with the corresponding time-matched depth frames for each mouse color were selected for annotation. The frames were manually chosen to ensure variety of mouse posture and position throughout the cage. Of each set of one hundred images, only seventy-five were selected for training and validation and the remaining 25 were set aside for testing. Of the 75 images in the training set, 95 percent (i.e., 95% of the 75 images) was used for testing, and the other five percent for validation. DeepLabCut was then used to train a Resnet-50 model on the depth images corresponding to the annotated RGB frames. The net was run for 182,000 iterations with a batch size of one. The trained model was then tested using a set composed of 25 images from each color mouse.

To provide grounds for establishing keypoint detection accuracy, a subset of 200 images was labelled by two separate annotators. The dually annotated set was then used to compute an object keypoint similarity (OKS) metric^11^. The keypoints detection accuracy, as based on the OKS measure, for depth images were scrutinized using the described test set. The trained DLC model was used for inference on the annotated test datasets. The detection accuracy measure for the predictive keypoint detection models was calculated based on the OKS determined previously. The OKS for the 75 depth images was computed. The computed average OKS for all depth images had an average OKS of 0.964. Computing the average OKS for each coat color separately yielded 0.963, 0.963, 0.966 for black, white, and tan respectively.

**Figure 11:**
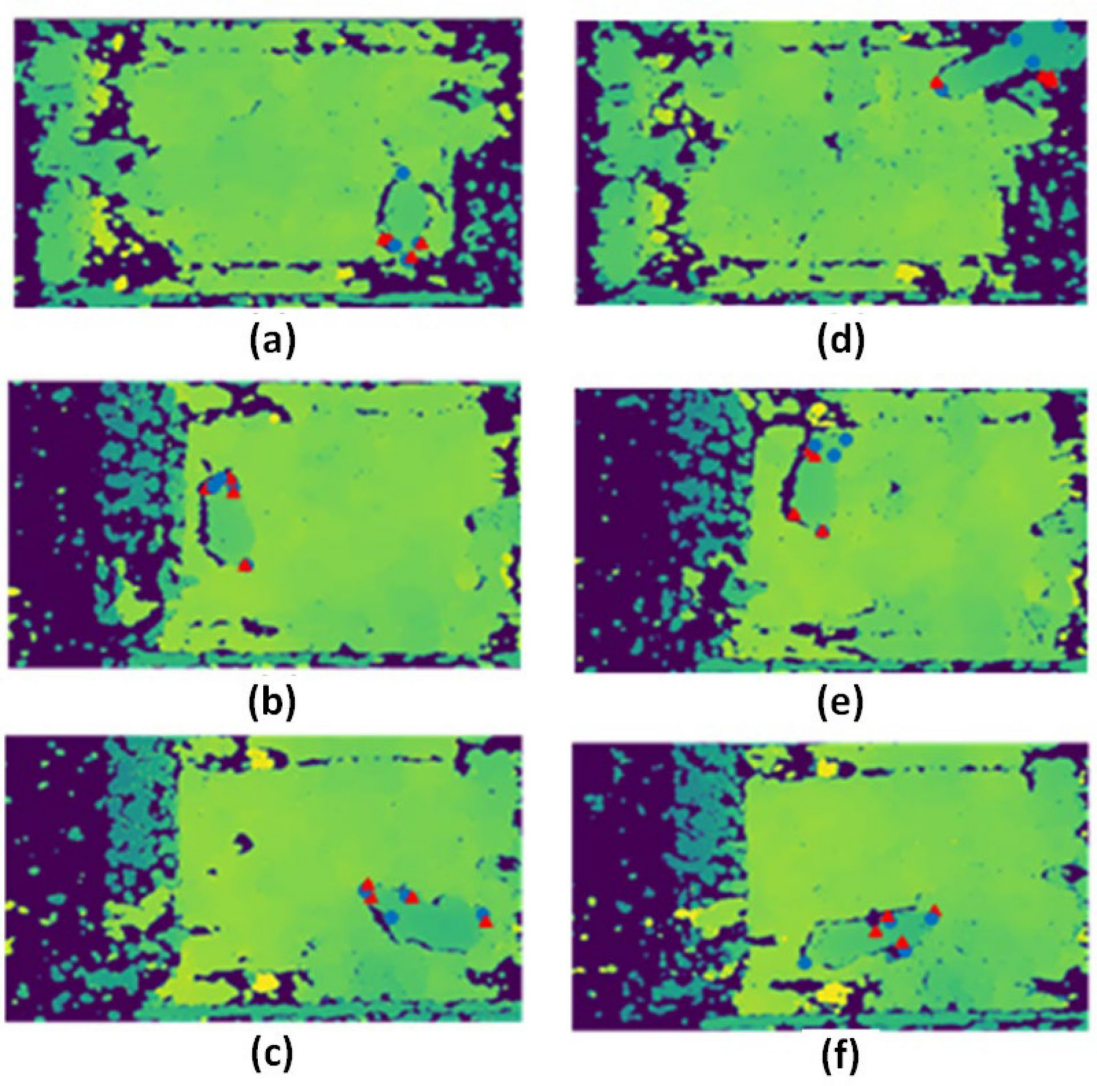
The best OKS values for white, black and tan depth frames are shown in (**a**, **b**, **c**). The worst OKS values for white, black, and tan depth frames are shown in (**d**, **e**, **f**). The ground truth annotations are labeled with a blue circle while the model’s annotations are labeled with a red triangle.

Figure 11 shows the images with ground-truth and with predictions overlaid on depth images, i.e., those used for inference, for highest and lowest OKS results for white, black and tan mice receptively.

## Discussion

We successfully developed an open-source system for mouse home-cage monitoring. With access to fabrication tools, such as 3D printers and laser cutters, one can utilize the CAD files and refer to the detailed instructions on the Github page to duplicate the system. The mechanical assembly includes a modified feeding hopper that was injection molded. The modified hopper takes up usable cage floor space reducing the usable living space in each cage from a maximum housing capacity of five adult mice to three. Video acquisition can be accomplished via one of several options outlined in the Results section. The combined system gives researchers a low-cost option to conduct monitoring experiments. Thorough assembly instructions are given on the project’s Github page (https://github.com/NIH-CIT-OIR-SPIS/MouseVUER).

The system can be integrated in an Allentown NexGen rack. Operation in a ventilated rack is perhaps the most desirable, and thus significant design considerations were made to achieve integration with the ventilated rack. Integrating video monitoring into ventilated racks allows researchers to measure behavior continuously and automatically for mice during experiments without disrupting animal husbandry procedures. Operation within ventilated racks also enables wider usage of the system, including animal well-being monitoring that is typically done by care staff such as disease outbreak and birth/death events. Integration in the ventilated rack, however, is greatly hindered by rack and cage obstacles. Design choices were made to circumvent the mechanical obstacles. The ideal solution would be redesigning racks and cages with video monitoring integration in mind. Until such designs become more available by rack manufacturers, a high-quality yet easy to assemble and install video system retrofit into an existing rack would offer scientists and animal care professionals quantitative insight into the activity of mice under experiment. The system can potentially serve to highlight the challenges of integration and give perspective on what can be gained by in-rack automated monitoring. We note, however, that the MouseVUER system is only compatible with the Allentown NexGen rack. Other commercial racks would present different design challenges that would almost certainly not be addressed by the MouseVUER system. We also note that when the system is used in the Allentown rack, it reduces the storage capacity to 50% since the camera enclosure takes up the cage slot above the cage to be monitored. To allow usage outside Allentown racks, we have designed a non-ventilated rack that would serve as compact housing for MouseVUER units.

An effective video acquisition solution should feature: (a) reliable streaming to a local computer or to a remote processing server, (b) efficient video compression to reduce streaming bandwidth and storage requirements, and (c) user-friendly means of specifying recording parameters such as start/stop times and experiment metadata. Experiment metadata could include cage information (e.g., age, gender, and strain of mouse) as well as trial data (e.g., trial name and experimenter name). As mentioned in the Results section, the MouseVUER system includes a video acquisition with the aforementioned features. Aivero’s software offers a commercial solution with more enhanced user experience and better compression ratios that was obtained with our software.

The MouseVUER system design we have developed successfully utilizes depth imaging technology for home-cage monitoring. The data presented in the Results section shows that one can accurately predict keypoints for the depth images, despite the colorized depth images being less visually pleasing than RGB images. Depth images provide a clear advantage over 2D images, namely that the 3D position of the keypoint is inferred as opposed to the 2D position as in conventional single camera video. That is clearly because the 2D predicted keypoint position in the depth image has a depth value associated with it. Therefore, with camera calibration information, one can compute a 3D physical position of the keypoint.

While there are several open-source tools for processing video, we hope that the open-source cage design will result in open-source processing and analysis tools specific to MouseVUER, capitalizing on what sets it apart from other systems including the depth video streaming capability. The overall open-source nature of the system should greatly enhance the reproducibility of results, a big concern for scientists. All the commercial systems are based on conventional 2D video, and all have associated processing software with different capabilities. Despite their capabilities and accessibility, the high cost of commercial systems limits their widespread use in research. Additionally, proprietary algorithms can hinder reproducibility of results in other laboratories that are not in possession of the same commercial system. An open-source home-cage monitoring system along with open-source video analysis tools would facilitate the reproducibility of research results while eliminating the high cost of commercial systems.

## Data Availability

All data generated or analysed during this study are included in this published article. In addition, all design files and software are included on the project Github site (https://github.com/NIH-CIT-OIR-SPIS/MouseVUER).
